# Retraction: Corrigendum: The heart of silk road “Xinjiang”, its genetic portray, and forensic parameters inferred from autosomal STRs

**DOI:** 10.3389/fgene.2024.1399880

**Published:** 2024-03-19

**Authors:** Frontiers Editorial Office

Following publication of this article, ethical concerns were brought to the attention of Frontiers. Some concerns were rectified through this Corrigendum, however during subsequent examination of documentation provided by the authors post-publication, additional significant issues with the ethical approval were discovered.

Frontiers engaged with the ethics committee responsible for reviewing and approving the study. The ethics committee was unable to confirm that they had reviewed and approved the study in question.

Due to the remaining ethical concerns and the inability to verify the validity of the ethical approval for this study, we are retracting this publication.

This retraction was approved by the Chief Editor of Frontiers in Genetics and the Editor-in-Chief of Frontiers. The authors do not agree to this retraction.

